# Determinants of a healthy lifestyle and use of preventive screening in Canada

**DOI:** 10.1186/1471-2458-6-275

**Published:** 2006-11-07

**Authors:** Vikky Qi, Susan P Phillips, Wilma M Hopman

**Affiliations:** 1 Department of Family Medicine, Queen's University, Kingston, Ontario, Canada; 2 Clinical Research Centre, Kingston General Hospital; Department of Community Health and Epidemiology, Queen's University, Kingston, Ontario, Canada

## Abstract

**Background:**

This study explores the associations between individual characteristics such as income and education with health behaviours and utilization of preventive screening.

**Methods:**

Data from the Canadian National Population Health Survey (NPHS) 1998–9 were used. Independent variables were income, education, age, sex, marital status, body mass index, urban/rural residence and access to a regular physician. Dependent variables included smoking, excessive alcohol use, physical activity, blood pressure checks, mammography in past year and Pap smear in past 3 years. Logistic regression models were developed for each dependent variable.

**Results:**

13,756 persons 20 years of age and older completed the health portion of the NPHS. In general, higher levels of income were associated with healthier behaviours, as were higher levels of education, although there were exceptions to both. The results for age and gender also varied depending on the outcome. The presence of a regular medical doctor was associated with increased rates of all preventive screening and reduced rates of smoking.

**Conclusion:**

These results expand upon previous data suggesting that socioeconomic disparities in healthy behaviours and health promotion continue to exist despite equal access to medical screening within the Canadian healthcare context. Knowledge, resources and the presence of a regular medical doctor are important factors associated with identified differences.

## Background

A socioeconomic gradient favouring those with greater income exists for a variety of chronic conditions, including cardiovascular disease, most cancers, diabetes, hypertension, arthritis, respiratory disease, gastrointestinal disease and metabolic syndrome [1]. Many of these can be prevented, identified early, or ameliorated by screening and individual behaviour.

Major behavioral factors affecting health include nutrition, physical activity, tobacco use, and excessive alcohol consumption. Low socioeconomic status has been associated with a more sedentary lifestyle and lower fruit and vegetable consumption [2]. A study based on the 1990 Canadian Health Promotion Survey found significant associations between socioeconomic class and patterns of exercise [3]. Although patterns of alcohol consumption are more complex, low socioeconomic status is linked to heavy drinking [2,4], and tobacco smoking follows the same trend [2,3,5-7].

Screenings such as mammography, Papanicolaou (Pap) smear testing and blood pressure (BP) measurement reduce mortality [8-10]. Studies in the US show that greater wealth is associated with higher utilization of these screening tests [11-15], even after controlling for insurance status, health status and usual source of care [16]. Likewise, a study comparing socioeconomic disparities in breast and cervical cancer screening in Ontario and the US found that the association between income and screening was similar in both populations, suggesting that factors unrelated to medical insurance coverage and access to care play a significant role [11].

Lifestyle factors and screening are components of health promotion and prevention. The Canadian Task Force on Preventive Health Care recommends that physicians counsel patients on nutritional habits, physical activity, smoking cessation and alcohol abuse, and offer blood pressure checks for all adults, Pap smears every 1–3 years for women from first sexual activity to age 69, and mammograms every 1–2 years for women aged 50–69 [6].

Previous studies in Canada have been based on earlier data, were focused on specific behaviors and maneuvers, or were limited to specific areas of the country [11-13]. The purpose of this study is to gain a more current view of the association between socioeconomic status, as indicated by income and education, and health promotion, lifestyle factors and use of screening, using data from a representative population-based health survey.

## Methods

Data from the Health File of the 1998–9 National Population Health Survey (NPHS) household component were used. The survey included questions relating to socio-demographic information, such as age, sex, education, household income and ethnicity, as well as health status, determinants of health and use of health services. The household component of the NPHS sampled household residents of all provinces in Canada, excluding Indian reserves, Canadian Forces Bases and some remote areas. Within each household, information was collected from each member (general file) and one person was randomly selected to complete a more in-depth interview and a longitudinal follow-up (health file) [16]. In addition, a cross-sectional sample was selected to compensate for sample attrition since 1994–5, and to account for populations not represented by the initial sample and those who were born or immigrated after 1994.

The survey was conducted by computer assisted interviewing (CAI), which allowed interviews to be customized to each respondent. Interviewers were trained specifically on the use of CAI for this survey. Most interviews were conducted by telephone. Personal visits were made if respondents did not have telephone access or at the respondent's request. On average, the interview's duration was one hour per household.

Weights were computed using an approach where an initial weight representing the inverse probability of selection was computed, then adjusted to take into account the various specifics of the survey. The typical adjustment was to compensate for non-response; homogeneous response groups were formed based on data available from both respondents and non-respondents [16]. A detailed description of the randomized study selection, calculation of weights and data collection is available elsewhere (NPHS User Guide) [16]. Survey design variables such as the primary sampling unit were not included in the analysis, as these are incorporated into the sampling weights.

The principal independent variables of interest were total annual household income and highest level of education. Income groups were collapsed to form 5 categories: < $20,000, $20,000–39,999, $40,000–59,999, $60,000–79,999, and = $80,000 (all in Canadian dollars). Education was categorized as less than secondary school, secondary school graduate, other postsecondary (e.g. trade, technical or vocational school), and college or university. Other independent variables were age (represented in 5-year increments), sex (1 = male, 2 = female), marital status (1 = with a partner, 2 = no partner), body mass index (BMI, defined as weight in kilograms divided by height in meters squared) and residence (1 = rural, 2 = urban, where urban areas are defined as continuously built-up areas having a population concentration of 1,000 or more and a population density of 400 or more per square kilometer). In addition, participant responses to the question "Do you have a regular family doctor?" were also included as an independent variable (1 = yes, 2 = no). These covariates were included based on previous studies showing their relationship to health promotion and preventive services [17-21].

All dependent variables were dichotomous and included Pap smear within the past 3 years, mammogram within the past year, BP taken within the past year, smoking status (no versus daily/occasionally) and alcohol consumption = 12 drinks per week. Physical activity was based on responses to a number of questions, including 21 specific activities and the frequency with which the respondent engaged in these activities. These responses were used to develop an overall frequency indicator of "regular", "occasional" and "infrequent", which were collapsed into the dichotomous variable of "regular" versus "occasional/infrequent". All variables were categorized according the Canadian Task Force on Preventive Health Care recommendations [10]. For the Pap smear, analysis was conducted on the subgroup of women aged 20–69 years, and for mammography, the subgroup of women aged 50–69 years. The study was limited to adults aged 20 years and over, as a number of the variables did not apply to those under 20 years. Ethics review was not required for this study.

### Statistical analysis

Crude frequencies and adjusted frequencies based on the population weights were calculated. Independent associations of education and total household income with each dependent variable, adjusting for age, sex, marital status, body mass index (BMI), regular medical doctor, and rural versus urban residence, were assessed by logistic regression, with all variables entered simultaneously. Income and education were categorical variables, so separate estimates are presented for each level. However, age, beginning with 20 to 24 years and ending at 80+ years, was treated as continuous despite being ordinal in nature, as there were 13 levels. Separate regressions were carried out for each health prevention measure, and reported results include the odds ratio (OR) and the associated 95% confidence interval (CI). Missing values and those considered "not applicable" were excluded from the analysis, with the exception of income level, where this was added as a separate category as this represented 6% of the sample. Appropriate sampling weights were applied in accordance with the design of the NPHS [16]. All analyses were performed using SPSS [Version 12.0.1, SPSS Incorporated, Chicago, Illinois, 2003].

## Results

A total of 13,756 adults aged 20 years and over completed the health portion of the NPHS 1998–9 cycle. The response rate for the health portion was 82.6% overall, calculated as the number of health surveys completed/total number of selected households. Table 1 contains the raw and weighted values of the sample characteristics. Table 2 contains the frequencies and the weighted and unweighted percentages for each of the outcomes. Tables 3 and 4 provide the complete logistic regression model for each health behaviour, including the odds ratio and the 95% confidence intervals, as well as the significance level of each variable. The Cox & Snell r^2 ^provides an estimate of the percentage of variation in outcome accounted for by the model, while the Hosmer & Lemeshow X^2 ^provides an estimate of model fit (a significant p-value suggests that the model does not adequately fit the data).

### Blood pressure check

Lower (< $40,000) levels of income were associated with blood pressure checks, while the middle and higher ranges of income were not. Those whose level of income was not reported also had higher rates of blood pressure checks. On the other hand, those with lower education were less likely to have blood pressure checks. Other factors associated with a greater likelihood of having regular checks included older age, being female, urban residence, having a regular medical doctor, and higher BMI. Marital status was not significantly associated with blood pressure checks. The model accounted for 6.9% of the variation in outcome, although the goodness of fit tests suggests that the model does not adequately fit the data (p = 0.02).

### Mammography

In women aged 50–69, mammography within the past year was associated with income only for those in the mid- to high-income category, that is, between $60,000 and $79,000 annually. Education was not strongly associated with mammography. Other factors associated with higher rates of mammography included older age, having a regular medical doctor, and higher BMI. There was a trend toward a direct association between having a partner and mammography screening. Rural or urban residence was not significantly associated with mammography. The model accounted for 5.6% of the variation in outcome, and is a reasonable fit for the data (p = 0.13).

### Papanicolaou test

In women aged 20 to 69 years, there is a clear trend towards increased utilization of Pap testing as income increases, although the highest probability of being tested was in those whose income was not reported. Lower education was associated with lower use of Pap testing. Other covariates associated with a Pap test in the past 3 years included younger age and having a regular medical doctor. Marital status, rural or urban residence and BMI were not strongly associated with Pap testing. The model accounted for 7.9% of the variation in outcome, and is a reasonable fit for the data (p = 0.23).

### Smoking

Lower levels of income were associated with a greater likelihood of smoking. The lowest odds ratio for smoking was in those whose income was not reported. As education level increased, the odds of smoking decreased. Other covariates associated with smoking included lower age, marital status (no partner), no regular medical doctor, and lower BMI. There was a strong trend towards more smoking among men (p = 0.075). Rural versus urban residence was not significantly associated with smoking. The model accounted for 5.6% of the variation in outcome, and is a reasonable fit for the data (p = 0.13).

### Alcohol

Excessive alcohol consumption was more prevalent amongst those with lower incomes, but the association was only significant for the income level of $20,000–$39,000. Education level showed the same pattern, with higher odds of excessive drinking at lower education levels. Other factors associated with excessive drinking included older age, being male, and higher BMI. Marital status, rural versus urban residence and having a regular medical doctor were not strongly associated with alcohol consumption. The model only accounted for 2.6% of the variation in outcome, although the model was a reasonable fit for the data p = 0.25).

### Regular physical activity

Respondents with higher incomes were more likely to engage in regular physical activity. This association attained statistical significance in those with an income of greater than $80,000 annually. The group that did not report their income also had higher odds of being involved in regular physical activity. Lower education was associated with a lower likelihood of being involved in regular physical activity. Other factors associated with regular physical activity included being female, having no partner, urban residence and lower BMI. Age and presence of a regular medical doctor were not significantly associated with regular physical activity. The model was not strong, accounting for only 2.3% of the variation, but it was a very good fit for the data (p = 0.94).

## Discussion

Our results are compatible with previous studies, both in Canada and other countries. Several studies in Canada have linked mammography and Pap smear rates to socioeconomic status [11-13,22,23]. Another study in Canada linked socioeconomic status to patterns of exercise [3]. Lower income and education have been associated with smoking, alcohol consumption and blood pressure monitoring in U.S. and European studies [4,6,7].

Those with a higher household income were more likely to have had a Pap smear in the preceding three years, a mammogram in the previous year, to have lower levels of alcohol consumption, to be non-smokers, and to be physically active. However, those with lower incomes were more likely to have had their blood pressure checked in the previous 12 months, which may be explained by two factors. First, lower income Canadians use primary health care more frequently than their wealthier counterparts [2], affording more opportunities for physicians to check blood pressure. Secondly, lower income is associated with higher rates of hypertension, diabetes, heart disease and cerebral vascular disease [1], so blood pressure may have been monitored as a part of disease management rather than as a preventive screening.

Increased income was also associated with decreased alcohol consumption. Although previous studies have shown conflicting results, moderate drinking appears to be more common at higher socioeconomic levels, and heavy drinking more common at lower socioeconomic levels [4,5], findings supported by our results.

Higher educational level was associated with a BP check in the past year, not smoking, not drinking excessively and increased physical activity. Women with less education were also less likely to have had a Pap smear in the previous three years. There was no strong association between education and mammography.

Prerequisites for regular physical activity include facilities, motivation and time. Those with a lower income and education may have limited means to pursue healthy preventive behaviours, as poorer neighborhoods often lack good recreational facilities and those with a lower income are less able to afford memberships or equipment for physical activity.

Access to a regular medical doctor was significantly associated with a reduced rate of smoking, as were higher income and higher education. One study showed that rates of smoking initiation are similar regardless of education, but quitting rates are dramatically higher in the educated [2]. The impact of access to medical care can also be seen in the higher rates of screening and use of preventive health services among those with a regular family physician, as well as the lower rate of smoking mentioned above. In Canada, the number of physicians per 1,000 population has decreased from 1.91 in 1995 to 1.85 in 1999. Projections based on current supply levels suggest this ratio will decrease to 1.4/1000 in 2021 [24]. With 13.5% of respondents indicating that they had no regular family physician, this can have a serious detrimental effect on the health of Canadians, and the use of preventive heath screening and services.

Limitations of this study should be discussed. The NPHS data were based exclusively on self-reporting. Thus, some of the measures of preventive health may have been over- or underestimated. In addition, the large sample size, while being a strength of the NPHS, also means that small differences can attain statistical significance. For this reason, the OR and the 95% CI were provided, to allow the application of clinical judgment when interpreting the results. The study excludes certain groups, such as Aboriginals, military personnel and those who spoke neither English nor French. Because of the nature and length of the interview, the results may be skewed towards those who had time to respond to the questions. As the data are cross-sectional in nature, associations can be identified but direction and causality cannot be inferred. Finally, it would be of interest to examine these associations within men and women separately, in future work.

## Conclusion

Lower socioeconomic status has consistently been linked to chronic disease and mortality, while certain lifestyle factors and preventive health strategies improve health. This study explored the associations of income and education with lifestyle factors such as smoking, alcohol use and exercise, as well as with utilization of preventive screening. The findings support and expand on those of previous research. Factors contributing to identified differences include knowledge, resources and availability of physicians. Despite equal access to medical screening and intervention within the Canadian health care context, socio-economic disparities persist. Targeted interventions would likely contribute to reducing these disparities in the health of Canadians.

## Competing interests

The author(s) declare that they have no competing interests.

## Authors' contributions

VQ and SPP participated in the conception and design of the study; VQ and WMH were involved in the analysis; VQ, SPP and WMH were involved in the interpretation of the findings and the drafting of the manuscript; all have read and approved the final manuscript.

## Pre-publication history

The pre-publication history for this paper can be accessed here:



## Figures and Tables

**Table 1:** Sample Characteristics and Frequencies (n = 13,756)

Variable	Number	Percent	Weighted percent
**Total Household Income**			
< $20,000	2879	20.9	15.6
$20,000–39,999	3805	27.7	25.1
$40,000–59,999	2776	20.2	21.6
$60,000–79,999	1712	12.4	14.7
>= $80,000	1762	12.8	16.5
Missing	822	6.0	6.5

**Education Level**			
< Secondary School	3505	25.5	22.5
Secondary School Graduate	2005	14.6	15.4
Other Post-Secondary	3575	26.0	26.7
College/University Degree	4659	33.9	35.3

Age Group*			
20–39 years	5328	38.8	40.8
40–59 years	4740	34.5	37.0
60–79 years	3021	22.0	19.1
>= 80 years	667	4.8	3.2

**Sex**			
Male	6248	45.4	48.8
Female	7508	54.6	51.2

**Marital Status**			
With a partner	8198	59.6	65.2
Without a partner	5558	40.4	34.8

**Rural Versus Urban Residence**			
Rural	3058	22.2	16.7
Urban	10697	77.8	83.3

**Regular Medical Doctor**			
Yes	11956	86.9	86.5
No	1799	13.1	13.5

**Body Mass Index (mean ± standard deviation)**	10683	25.8 ± 4.6	25.6 ± 4.5

12 were missing education level; 1 was missing information about regular doctor

* In the analyses, age was categorized in 5-year increments, e.g. 20–24, 25–29, etc.

**Table 2:** Preventive Health Characteristics and Frequencies

**Preventive Health Measure**	**Total**	**Percent**	**Weighted Percent**
Blood pressure check in past year	10298	77.7	76.6
Mammogram in past year in women aged 50 – 69	869	54.7	55.3
Pap Smear in past 3 years in women aged 20 – 69	4918	85.1	81.8
Regular smoker	4252	30.9	27.7
Regularly drinks > 12 units of alcohol per week	368	2.7	2.0
Frequent physical activity	8104	58.9	61.1

**Table 3:** Logistic Regression Models for Blood Pressure, Mammogram and Pap Test

**Blood Pressure Check in Past Year, N = 10,320** **Cox & Snell r^2 ^= 0.069** **Hosmer & Lemeshow Χ^2 ^= 18.3, p = 0.02**	**Odds Ratio (95% CI)**	**p-value**
Total household income (reference = < $19,999)		
$20–39,999	1.42 (1.12, 1.79)	0.003
$40–59,999	1.14 (0.92, 1.41)	0.245
$60–79,999	1.11 (0.89, 1.38)	0.374
$80,000+	1.14 (0.90, 1.44)	0.267
Missing	1.33 (1.06, 1.68)	0.016
Education level (reference < secondary school)		
Secondary school graduate	0.83 (0.72, 0.95)	0.008
Other post-secondary	0.83 (0.72, 0.96)	0.009
College/University degree(s)	1.00 (0.89, 1.13)	0.961
Age (5-year increments)	1.12 (1.09, 1.14)	<0.001
Sex (1 = male, 2 = female)	1.86 (1.69, 2.04)	<0.001
Marital status (1 = with partner, 2 = no partner)	0.97 (0.88, 1.08)	0.621
Rural/Urban (1 = rural, 2 = urban)	1.14 (1.02, 1.27)	0.024
Regular medical doctor (1 = yes, 2 = no)	0.40 (0.36, 0.45)	<0.001
Body mass index (numeric)	1.05 (1.04, 1.06)	<0.001

**Mammogram in Past Year, Women 50–69, N = 1,192** **Cox & Snell r^2 ^= 0.056** **Hosmer & Lemeshow Χ^2 ^= 12.6, p = 0.13**	**Odds Ratio (95% CI)**	**p-value**

Total household income (reference = < $19,999)		
$20–39,999	1.01 (0.58, 1.76)	0.963
$40–59,999	1.28 (0.77, 2.13)	0.343
$60–79,999	1.90 (1.11, 3.25)	0.019
$80,000+	1.15 (0.63, 2.08)	0.652
Missing	1.59 (0.89, 2.83)	0.114
Education level (reference < secondary school)		
Secondary school graduate	0.85 (0.62, 1.18)	0.339
Other post-secondary	1.03 (0.71, 1.51)	0.865
College/University degree(s)	0.89 (0.65, 1.22)	0.480
Age (5-year increments)	1.16 (1.00, 1.34)	0.047
Marital status (1 = with partner, 2 = no partner)	0.80 (0.60, 1.05)	0.106
Rural/Urban (1 = rural, 2 = urban)	1.14 (0.85, 1.520	0.377
Regular medical doctor (1 = yes, 2 = no)	0.53 (0.29, 0.97)	0.040
Body mass index (numeric)	1.02 (1.00, 1.05)	0.059

**Pap Test in Past 3 Years, Women 20–69, N = 5,192** **Cox & Snell r^2 ^= 0.079** **Hosmer & Lemeshow Χ^2 ^= 10.5, p = 0.23**	**Odds Ratio (95% CI)**	**p-value**

Total household income (reference = < $19,999)		
$20–39,999	0.85 (0.57, 1.25)	0.406
$40–59,999	1.15 (0.80, 1.68)	0.450
$60–79,999	1.27 (0.86, 1.86)	0.226
$80,000+	1.59 (1.04, 2.42)	0.033
Missing	2.28 (1.47, 3.56)	<0.001
Education level (reference < secondary school)		
Secondary school graduate	0.73 (0.58, 0.93)	0.011
Other post-secondary	1.11 (0.84, 1.46)	0.458
College/University degree(s)	0.93 (0.75, 1.15)	0.501
Age (5-year increments)	0.76 (0.73, 0.79)	<0.001
Marital status (1 = with partner, 2 = no partner)	1.06 (0.87, 1.30)	0.545
Rural/Urban (1 = rural, 2 = urban)	1.09 (0.89, 1.32	0.425
Regular medical doctor (1 = yes, 2 = no)	0.36 (0.28, 0.46)	<0.001
Body mass index (numeric)	0.99 (0.97, 1.00)	0.142

CI = Confidence Interval

**Table 4:** Logistic Regression Models for Regular Smoking, Alcohol Consumption and Physical Activity

**Regular Smoker, N = 10,664** **Cox & Snell r^2 ^= 0.056** **Hosmer & Lemeshow Χ^2 ^= 12.6, p = 0.13**	**Odds Ratio (95% CI)**	**p-value**
Total household income (reference = < $19,999)		
$20–39,999	1.40 (1.14, 1.72)	0.002
$40–59,999	1.32 (1.09, 1.61)	0.005
$60–79,999	1.07 (0.88, 1.31)	0.496
$80,000+	0.96 (0.77, 1.18)	0.682
Missing	0.62 (0.50, 0.77)	<0.001
Education level (reference < secondary school)		
Secondary school graduate	2.59 (2.29, 2.92)	<0.001
Other post-secondary	1.71 (1.51, 1.94)	<0.001
College/University degree(s)	1.45 (1.31, 1.61)	<0.001
Age (5-year increments)	0.97 (0.95, 0.98)	<0.001
Sex (1 = male, 2 = female)	0.93 (0.85, 1.01)	0.075
Marital status (1 = with partner, 2 = no partner)	1.16 (1.06, 1.28)	0.001
Rural/Urban (1 = rural, 2 = urban)	0.93 (0.84, 1.02)	0.130
Regular medical doctor (1 = yes, 2 = no)	1.25 (1.12, 1.40)	<0.001
Body mass index (numeric)	0.98 (0.97, 0.99)	<0.001

**Alcohol Consumption > 12/wk, N = 10,677** **Cox & Snell r^2 ^= 0.026** **Hosmer & Lemeshow Χ^2 ^= 10.3, p = 0.25**	**Odds Ratio (95% CI)**	**p-value**

Total household income (reference = < $19,999)		
$20–39,999	2.98 (1.46, 6.04)	0.003
$40–59,999	1.87 (0.93, 3.76)	0.081
$60–79,999	1.27 (0.61, 2.62)	0.520
$80,000+	1.15 (0.53, 2.47)	0.723
Missing	1.30 (0.61, 2.79)	0.498
Education level (reference < secondary school)		
Secondary school graduate	2.08 (1.47, 2.93)	<0.001
Other post-secondary	1.15 (0.74, 1.79)	0.527
College/University degree(s)	1.74 (1.24, 2.43)	0.001
Age (5-year increments)	1.23 (1.16, 1.30)	<0.001
Sex (1 = male, 2 = female)	0.22 (0.16, 0.29)	<0.001
Marital status (1 = with partner, 2 = no partner)	1.10 (0.83, 1.45)	0.508
Rural/Urban (1 = rural, 2 = urban)	0.95 (0.72, 1.25)	0.692
Regular medical doctor (1 = yes, 2 = no)	0.81 (0.56, 1.16)	0.245
Body mass index (numeric)	1.03 (1.00, 1.06)	0.033

**Regular Physical Activity, N = 10,537** **Cox & Snell r^2 ^= 0.023** **Hosmer & Lemeshow Χ^2 ^= 2.8, p = 0.94**	**Odds Ratio (95% CI)**	**p-value**

Total household income (reference = < $19,999)		
$20–39,999	0.86 (0.70, 1.05)	0.145
$40–59,999	0.98 (0.81, 1.18)	0.806
$60–79,999	1.08 (0.89, 1.31)	0.446
$80,000+	1.33 (1.08, 1.62)	0.007
Missing	1.65 (1.34, 2.04)	<0.001
Education level (reference < secondary school)		
Secondary school graduate	0.69 (0.62, 0.78)	<0.001
Other post-secondary	0.81 (0.71, 0.91)	<0.001
College/University degree(s)	0.90 (0.81, 0.99)	0.001
Age (5-year increments)	0.99 (0.97, 1.01)	0.104
Sex (1 = male, 2 = female)	1.09 (1.01, 1.18)	0.038
Marital status (1 = with partner, 2 = no partner)	1.17 (1.07, 1.28)	0.001
Rural/Urban (1 = rural, 2 = urban)	1.10 (1.00, 1.21)	0.047
Regular medical doctor (1 = yes, 2 = no)	0.92 (0.82, 1.03)	0.141
Body mass index (numeric)	0.98 (0.97, 0.99)	<0.001

CI = Confidence Interval
